# A comparison of diagnostic performance between two quantitative rapid fecal calprotectin assays in detecting active inflammatory bowel disease

**DOI:** 10.1371/journal.pone.0255974

**Published:** 2021-08-12

**Authors:** Jong-Mi Lee, Joo Hee Jang, Ji Hyeong Ryu, Jaeeun Yoo, Bo-In Lee, Seung-Jun Kim, Eun-Jee Oh

**Affiliations:** 1 Department of Laboratory Medicine, Seoul St. Mary’s Hospital, College of Medicine, The Catholic University of Korea, Seoul, Korea; 2 Department of Biomedicine & Health Sciences, Graduate School, College of Medicine, The Catholic University of Korea, Seoul, Korea; 3 Department of Laboratory Medicine, Incheon St. Mary’s Hospital, College of Medicine, The Catholic University of Korea, Seoul, Korea; 4 Department of Internal Medicine, Division of Gastroenterology, College of Medicine, The Catholic University of Korea, Seoul, Korea; Changhua Christian Healthcare System: Changhua Christian Hospital, TAIWAN

## Abstract

**Background:**

Fecal calprotectin (FC) is widely used for the diagnosis and monitoring disease activity of inflammatory bowel disease (IBD). Quantitative rapid assays can be a reliable alternative to the time-consuming assay. This study aimed to evaluate and compare the diagnostic performance of two quantitative rapid FC assays (Ichroma calprotectin, and Buhlmann Quantum blue).

**Methods:**

A total of 192 patients were included in this study; 84 patients with IBD (67 ulcerative colitis and 17 Crohn’s disease) and 108 patients with non-IBD. We compared quantitative FC levels in different disease statuses and evaluated the correlation between the FC results of the two FC kits. Diagnostic performances in predicting active IBD were evaluated in reference to different cut-off levels.

**Results:**

The FC levels in 45 patients with active IBD as defined by endoscopic score were significantly higher compared to the inactive IBD and other diseases (*P*<0.05). Although the two assays’ results correlated (r = 0.642, P < 0.001), a significant deviation was observed (y (Buhlmannn) = -45.2 +8.9X (Ichroma)). The Diagnostic performances in predicting active IBD were comparable as area under the curve (AUC), 0.812, cut-off, 50, sensitivity, 64.4%, and specificity, 85.0% for iChroma assay and AUC, 0.826, cut-off, 100, sensitivity, 84.4%, and specificity 61.9% for Buhlmann Quantum Blue assay. FC levels using a cut-off of > 250 μg/g confirmed 85.7% (iChroma) and 64.1% (Buhlmann) of active IBD patients.

**Conclusion:**

The results of the two rapid FC assays iChroma and Buhlmann showed a significant correlation, but the two test results were not interchangeable. With optimized cut-off values, rapid FC tests could be helpful in the diagnosis of IBD and differentiating active IBD from inactive or organic bowel disease.

## Introduction

Inflammatory bowel diseases (IBD) are chronic diseases caused by inflammation of the intestines include ulcerative colitis (UC) and Crohn’s disease (CD). It is difficult to differentiate between organic disease and functional gastrointestinal (GI) disorders including irritable bowel syndrome (IBS). Endoscopy and histological evaluation are the gold standards for IBD diagnosis. However, the procedures involved in the two approaches are invasive which is unnecessary in some cases and therefore not advisable. In addition, they have significant implications of cost and complications.

Calprotectin is a complex of calcium and zinc-binding protein mainly released from neutrophils during inflammation [1]. During the bowel inflammation, neutrophils migrate to the intestinal lumen where they excrete the calprotectin. Fecal calprotectin (FC) is the most promising noninvasive biomarker for neutrophilic intestinal inflammation and differential diagnosis between organic and functional bowel disease. The clinical value of fecal calprotectin assays in the diagnosis and assessment of endoscopic disease activities of IBD has been proven [2–9]. Currently, there are many commercial assays for the detection of FC including rapid point-of-care test (POCT) [7, 10]. Rapid immunochromatographic assay tests have the advantages of simplicity, rapid detection and do not require an expensive analyzer, making it an acceptable assay in a majority of laboratory settings. Quantum Blue Calprotectin (BÜHLMANN Laboratories AG Baselstr, Switzerland), one of the commercially available quantitative immunochromatographic rapid tests, has demonstrated good analytical and clinical performance in many studies and has proven to be a reliable alternative for the time-consuming enzyme-linked immunosorbent assay (ELISA) [9–14]. However, the optimal cut-off values vary across the existing studies, and Quantum Blue Calprotectin consists of two types of cartridges with a different assay range: the normal range (20–300 μg/g feces) and high range (100–1,800 μg/g feces), requiring clear clinical information to choose the appropriate cartridge [10]. The iChroma calprotectin (Boditech Med Inc, Korea) is a newly introduced fluorometric immunoassay for the quantitative determination of FC which has the advantages of a wider detection range (1–1,000 μg/g feces) and a short reaction time (<10 min). Therefore, we evaluated the performances of the two assays based on the endoscopic findings and clinical diagnosis, in addition to the head-to-head comparison. The aim of this study is to evaluate the diagnostic performance of iChroma calprotectin and its comparability with the commonly used Quantum Blue Calprotectin assay in distinguishing active IBD from other GI disorders including inactive IBD, IBS and conditions associated with GI symptoms.

## Materials and methods

### 1. Subjects

A total of 192 patients were included in this study; 84 patients with IBD (67 UC and 17 CD) and 108 patients with non-IBD. Stool samples were retrospectively selected from fecal calprotectin requested samples in Seoul St. Mary’s Hospital (Seoul, Korea), from July 2018 to April 2020. All the included patients underwent colonoscopy. The diagnosis was established on the basis of clinical symptoms, endoscopic findings, pathology, and radiology findings without calprotectin results. The disease activities were assessed using the Mayo endoscopic subscore (MES) and the Simple Endoscopic Score for Crohn Disease (SES-CD) for UC and CD, respectively. Inactive IBD state was defined as MES 0–1 and SES-CD 0–1. There were 45 patients with active IBD patients (30 UC and 15 CD) included 3 UC and 13 CD patient at diagnosis before treatment. The exclusion criterion was: uncertain diagnosis, incomplete colonoscopic findings, history of bowel resection, or regular intake of aspirin or NSAID. This study was approved by the Institutional Review Board at Seoul St. Mary’s Hospital (KC19DESI0717). Written informed consent was waived by the board because the current study was retrospective in nature using medical records and leftover samples.

### 2. Fecal calprotectin assays

#### 2.1 Stool samples

Stool samples were transported to the laboratory at room temperature and stored at 2–8°C prior to extraction. For comparison, we mainly used left-over fecal specimen stored at 4°C within a week or frozen samples stored at -20°C within 6 months. The stability of the stored frozen stool sample was verified, and comparable results were confirmed by comparing the level of fecal calprotectin at the time of collection and after storage. The fecal calprotectin levels were measured using both ichroma calprotectin and Buhlmann Quantum Blue Calprotectin tests. The median time interval from FC measurement to colonoscopy was 75 days (95% Confidence interval (CI); 32–108 days)

#### 2.2 ichroma calprotecin test

The ichroma calprotectin assay is a fluorometric immunoassay for the quantitative determination of calprotectin in fecal extract (10–2,000 μg/g). Kit contains extraction devices for accurate sampling the desired amount of fecal specimen. The fecal samples were mixed and extracted in a one-step process according to the manufacturer’s instructions. Specific amounts of fecal samples were collected using a spiral-shaped sampling stick. Then the stick was immersed in the extraction buffer tube and shaken vigorously. Briefly, three drops of the mixture sample were loaded into the sample well on the cartridge using the tip on the tube. After 10 minutes of incubation at room temperature, ichroma^TM^ II scanned the sample-loaded cartridge and the results were read on the display screen.

#### 2.3 Buhlmann Quantum Blue test

Buhlmann Quantum Blue assay is an immunochromatography assay for quantitative calprotectin measurement. Stool samples were extracted in a two-step process using CALEX® Cap Device, a commercially available fecal extraction device. Stool samples were collected using a sampling pin and vortexed with an extraction buffer for 30 seconds. Then the samples were left to settle down for 10 minutes. The supernatant was transferred into a tube and diluted at a ratio of 1:10 using extraction buffer and vortex. After equilibrating the sample for at least 5 minutes, 60μl of the diluted extract was poured into the sample loading port. After incubating for 15 minutes, the extract was analyzed using Quantum Blue Reader. Kit consists of two types of cartridges with a different assay range: the normal range (20–300 μg/g feces) and high range (100–1,800 μg/g feces). The characteristics of the two rapid assays are summarized in Table 1.

**Table 1:** Characteristics of two quantitative rapid assays for fecal calprotectin measurements.

	Ichroma Calprotectin	Buhlmann Quantum Blue
Assay principles	Fluorometric immunoassay	Immunochromatographic assay
Proposed cut-off (μg/g)	50	50
Measuring range (μg/g)	10–2,000	Cartridge 1; 30–300
		Cartridge 2; 100–1,800
Precision (CV) by manufacturer	< 10%	< 15%
Sample extraction Procedure	1 step (extraction)	2 steps (extraction and dilution)
Product storage	Room temperature (4–30°C)	Refrigerated storage (2–8°C)
Analyzer	Ichroma II	Quantum Blue Reader

### 3. Statistical analysis

Quantitative results are described as median with range or 95% CI. The results from the two rapid tests were compared using Passing and Bablok regression and Spearman’s rank correlation. Diagnostic performance for active IBD was evaluated using receiver-operating characteristic (ROC) curve and area under the curve (AUC). Diagnostic accuracies including sensitivity, specificity, Likelihood Ratio (LR+), and post-test probability were calculated using various cut-off levels (optimal cut-off, cut-offs at fixed specificity) and results intervals. Statistical analyses were performed using MedCalc (v.16.4.4). A *P* value of less than 0.05 was considered statistically significant.

## Results

### 1. Patients’ characteristics

A total of 192 patients were analyzed in this study; 67 UC and 17 CD and 108 non-IBD. Based on the endoscopic results, patients were further categorized into four groups: 45 active IBD (30 UC and 15 CD), 39 inactive IBD (37 UC and 2 CD), 44 IBS, and 64 other GI disease control patients. Of the 67 patients with UC, 30 patients had MES >2, 17 patients had MES 1, and 20 patients had MES 0. Significant inflammatory activity was defined by MES 2–3 of calprotectin. Of the 17 patients with CD, two patients showed inactive CD (SES-CD 0–1). Other gastroenteric disease controls included patients with non-specific GI symptom (n = 25), enterocolitis (n = 19), colon adenoma or polyp (n = 11), GI ulcer or bleeding (n = 9). Demographics and calprotectin levels (median with 95% CI) according to the clinical diagnosis and IBD states are shown in Table 2.

**Table 2:** Demographics and FC results of the study cases.

	No. of patients	Age, Median (range)	M/F	Ichroma Calprotectin (μg/g)	Buhlmann Quantum Blue (μg/g)
UC	67	43 (17–82)	45/22	15.9 (10.0–46.3)*	130.0 (100.1–157.9)
active	30			56.6 (12.7–214.8)	141.5 (123.4–303.3)
inactive	37			10.0 (9.9–18.7)	99.9 (99.9–141.8)
CD	17	20 (11–65)	11/6	259.3 (69.1–578.5)	631.0 (302.8–1796.1)
active	15			354.9 (94.0–594.3)	813.0 (469.5–1800.1)
inactive	2			41.1	109.5
IBS	44	28 (15–80)	27/17	10.0 (10.0–10.0)	37.0 (29.9–80.2)
Other	64	67 (17–90)	33/31	10.0 (10.0–11.0)	100.5 (62.1–136.6)

*median (95% CI)

### 2. Fecal calprotectin levels according to the disease status

**Fig 1:**
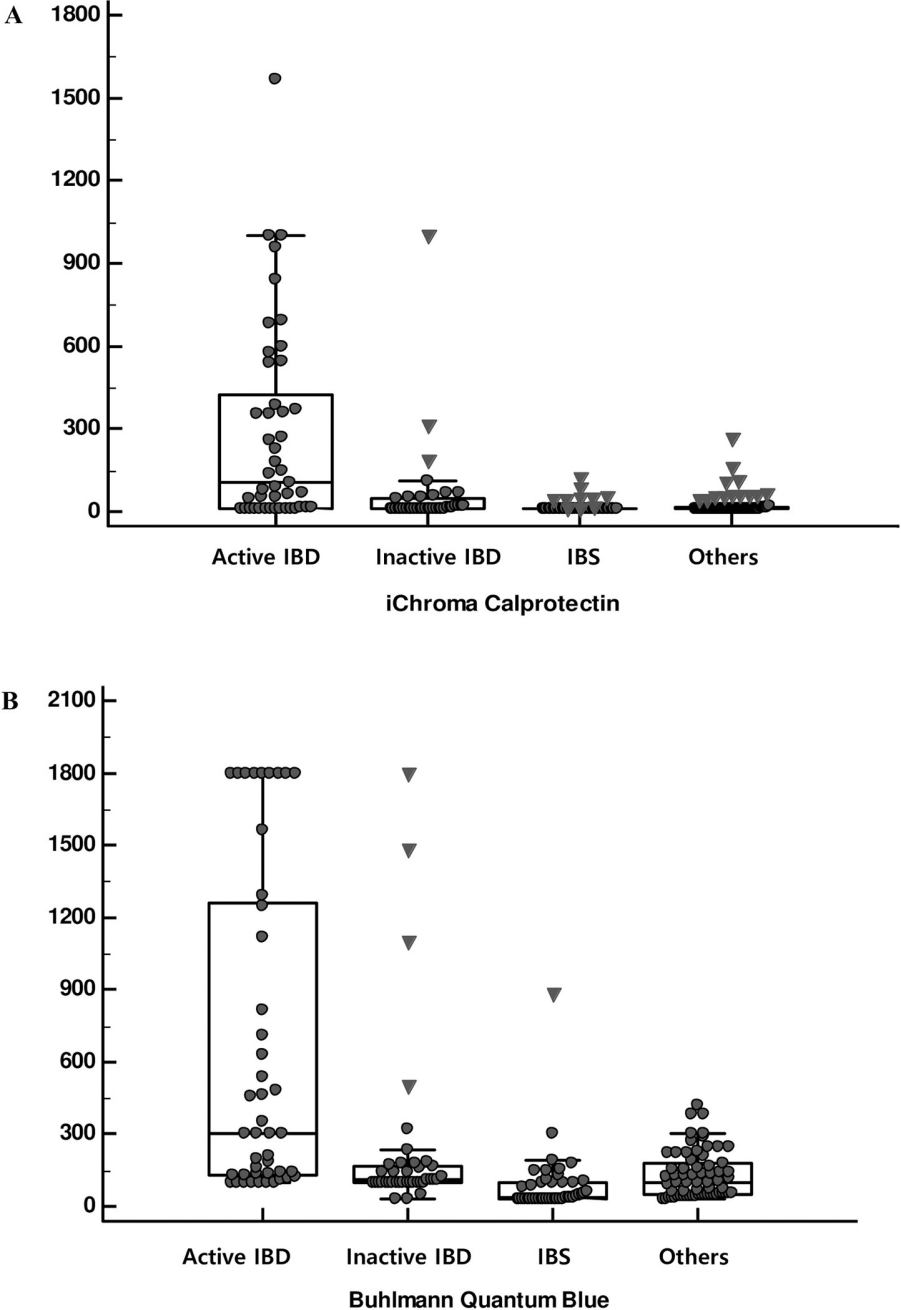
Fecal calprotectin levels by the two rapid tests according to the disease status. (A) iChroma calprotectin tests, (B) Buhlmann Quantum Blue tests.

FC results by the two rapid tests for the four groups (active IBD, inactive IBD, IBS, and others) were separately plotted in Fig 1. The median (95% CI) FC levels (μg/g) from iChromaCalptectin assay were 104.4 (49.0–315.1) in active IBD, 10.0 (10.0–18.8) in inactive IBD, 10.0 (10.0–10.0) in IBS and 10.0 (10.0–11.0) in others. Buhlmann Quantum Calprotectin assay results were 300.1 (150.8–587.7) in active IBD, 109.0 (99.9–140.2) in inactive IBD, 37.0 (29.9–80.2) in IBS and 10.5 (62.1–136.6) in others. Active IBD patients showed significantly higher FC levels (*P*<0.001) in both iChroma and Buhlmann FC assays compared to the inactive IBD or other diseases. There was no significant difference of FC levels among patients with inactive IBD, IBS, and other GI diseases in iChromaCalptectin assay (*P* = 0.071). On the other hand, FC levels from IBS patients were significantly lower than those from inactive IBS and other GI diseased by Buhlmann Quantum Calprotectin assay (P < 0.001). Of the 30 patients with active UC, 16 (53.3%) patients showed proctitis or proctosigmoiditis in colonoscopy and had lower FC levels (12.3 (10.0–55.3) μg/g) with iChroma Calprotectin assay compared to the other 14 active UC patients (311.8 (64.1–686.4) μg/g) (*P* < 0.001). There were no significant differences in Buhlmann Quantum Blue assay FC levels according to the disease location (135.5 (99.9–244.0) vs. 300.1 (121.8–826.3), *P* = 0.148). Nineteen patients with enterocolitis showed tendency of higher FC levels in both assays (143.0 (100.8–222.7) μg/g in Buhlmann Quantum Blue and 10.0 (10.0–16.8) μg/g in Ichroma Calprotectin), but it was not statistically significant (*P* = 0.074, *P* = 0.078).

### 3. Comparison of the two rapid FC assays

**Fig 2:**
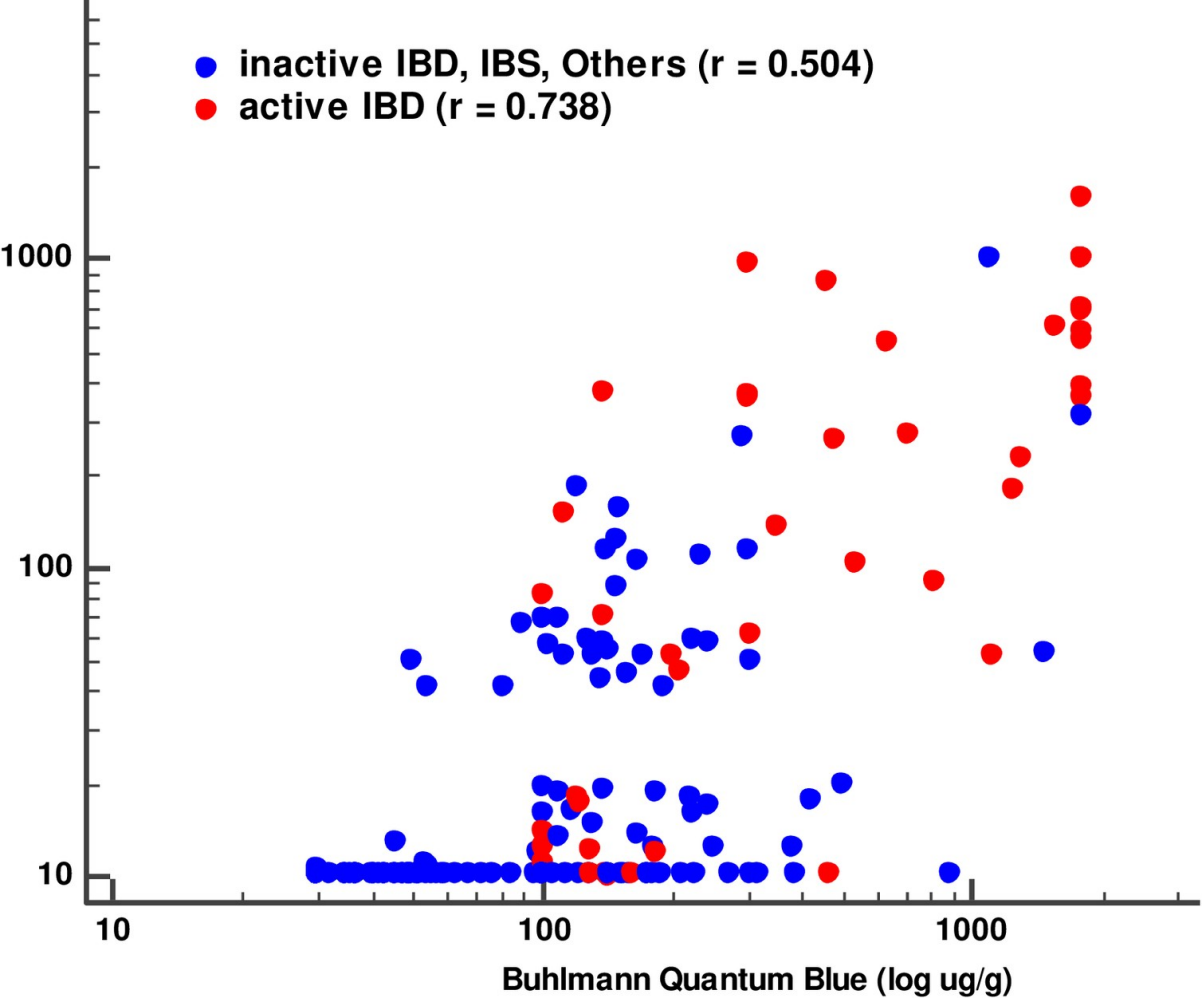
Correlation between fecal calprotectin results by iChroma calprotectin and Buhlmann Quantum Blue assays. The correlation was stronger in patients with active IBD (r = 0.738) than in the other groups (r = 0.504).

Correlation analysis between iChroma and Buhlmann Quantum Blue assays FC levels was performed. Spearman’s rank correlation coefficient was 0.642(95% CI, 0.550–0.718), and the FC results of the two assays correlated significantly (*P*< 0.001). Fig 2 is a scatter diagram of the two assays’ FC results presented according to the active IBD and the other groups (in active IBD, IBS, and other gastroenteric diseases). There was a stronger correlation between the two assays’ FC results for the active IBD patients (r = 0.738) compared to those in the other groups (r = 0.504). Although the two assays’ FC results correlated, a significant deviation was observed. The FC values measured with Buhlmann Quantum Blue assays were significantly higher compared to iChroma FC values (*P*< 0.05). In passing and Bablock regression analysis, the intercept and slope were 8.918 (95%CI; 5.363–21.401) and -45.2 (-179.8 - -5.1), respectively.

### 4. Diagnostic performance and overall accuracy of the two fecal calprotectin assays

**Fig 3:**
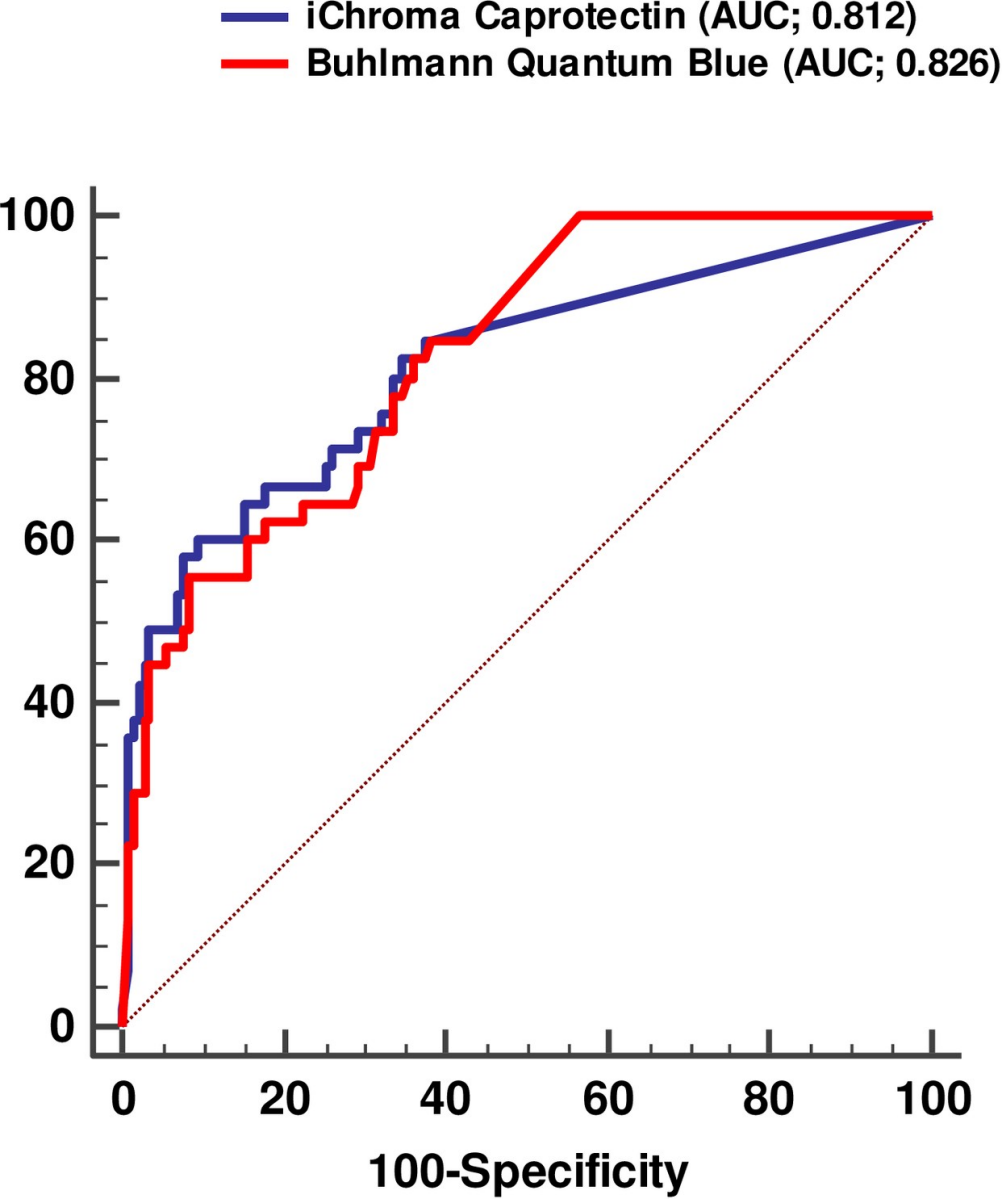
ROC curve analysis of iChroma calprotectin and Buhlmann Quantum Blue assays for detecting active IBD. The AUCs were 0.812 and 0.826 for iChroma and Buhlmann Quantum Blue, respectively.

The diagnostic performance including sensitivity and specificity for detecting active IBD for the two FC assays were calculated. The areas under the curves (AUC) were 0.812 (0.750–0.865) and 0.826 (0.765–0.877) for iChroma and Buhlmann Quantum Blue, respectively. There was no significant difference in the AUCs (*P*> 0.05) of the two assays (Fig 3). The diagnostic performance of the two assays predicting active IBD is summarized in Table 3 according to the different cut-off values and result intervals. At the cut-off value of 50 μg/g which was proposed by two manufacturers, the sensitivities of iChroma and Buhlmann Quantum Blue were 64.4% and 100%, and the specificities were 85.0% and <10.0%, respectively. Based on the ROC curve analysis, cut-offs at fixed specificity of 80% and 95% were 40.8 μg/g and 112.4 μg/g for iChroma assay, and 172.8μg/g and 372.5 μg/g for Buhlmann Quantum Blue, respectively. The sensitivities at these cut-off values were less than 70%, ranged 44.4–66.7% in both assays. LRs and post-test probabilities for chosen result intervals were calculated. Using a cut-off of > 250 μg/g iChroma Calprotectin assay confirmed 85.7% and Buhlmann Quantum Blue assays 64.1% of the active IBD patients.

**Table 3:** Diagnostic performance of the two rapid fecal calprotectin assays predicting active IBD according to the various cut-off values and result intervals.

	Cut-off (μg/g)	Sensitivity (95% CI)	Specificity (95% CI)	Result interval (μg/g)	Likelihood ratio (95% CI)	post-test probability
Ichroma Calprotectin	50	64.4 (48.8–8.1)	85.0 (78.2–90.4)	Negative	0.4 (0.3–0.6)	11.6%
	100	48.9 (33.7–64.2)	93.2 (87.8–96.7)	50–150	1.5 (0.7–3.0)	31.0%
	40.8*	66.7 (51.1–80.0)	80	150–250	3.3 (0.5–22.5)	50.0%
	112.4**	48.9 (31.1–64.4)	95	>250	19.6 (6.0–63.5)	85.7%
Buhlmann Quantum Blue	50	100	<10	Negative	0 (0.0–0.6)	0.0%
	100	84.4 (70.5–93.5)	61.9 (53.5–69.8)	50–150	0.8 (0.5–1.2)	19.8%
	172.8*	62.2 (46.7–75.6)	80	150–250	0.5 (0.2–1.5)	14.3%
	372.5**	44.4 (24.4–60.0)	95	>250	5.8 (3.3–10.2)	64.1%

*Cut-off at fixed specificity of 80%

** Cut-off at fixed specificity of 95%

95% CI, 95% CI

## Discussion

The use of quantitative immunochromatographic POCT for stool examination is a promising alternative to the time-consuming ELISA [15, 16]. Previous studies have shown Buhlman Quantum Blue POCT to have a reliable performance which was in agreement with an established ELISA in FC measurement [9–14]. The iChroma calprotectin is a new fluorometric immunoassay for quantitative determination of FC which has the advantages of a wider detection range (1–1,000 μg/g feces) and a short reaction time (<10 min). In terms of clinical efficiency, this assay benefits clinicians by covering low to high calprotectin levels in a single test before determining disease activity. This study aimed to evaluate and compare the diagnostic performance of the two rapid assays. We compared the results of the quantitative levels measurements of different disease statuses and evaluated the correlation between the results of the two FC kits. Finally, we analyzed the diagnostic performances in predicting active IBD according to various cut-off levels.

Based on our results, the FC levels by the two assays were significantly increased in the active IBD group. In correlation analysis, the results of the two assays result correlated significantly, especially in active IBD cases (r = 0.738). However, in regression analysis, Buhlmann Quantum blue revealed significantly higher FC values than Ichroma calprotectin (slope; 8.918). These differences could be attributed to the differences in standards, antibodies used in both kits, and immunoassay techniques from different manufacturers. This is contradicting because the cut-off levels proposed by the two manufacturers were the same-50.0 μg/g. These results are in agreement with prior studies in which Labaere et al., reported that Quantum blue’s FC results values were higher (up to 5 times) than those of ELISA or other immunochromatography assays, and Jang et al., reported low specificity (40%) of Buhlmann Quantum Blue assay in discriminating IBD from other colitis groups[11, 12]. The findings of the present study confirm that it is inappropriate to directly compare absolute calprotectin levels between different kits and it is necessary of kit-specific cut-off level [6]. This highlights the need for standardization of FC levels. In addition, clinical laboratories should establish or verify cut-off values according to their intended use and monitor patients using the same assay [7].

Several studies have assessed the predictive capacities of fecal calprotectin measurements towards endoscopic procedures and suggested different cut-off levels [8, 17, 18]. In this study, we evaluated the diagnostic performance of the two assays in predicting active inflammation according to different cut-off values. Using the manufacturer’s proposed cut-off value of 50 μg/g, Ichroma Calprotectin and Buhlmann Quantum Blue showed different diagnostic performances with sensitivities of 64.4% and 100%, and specificities 85.0% and <10.0%, respectively. The performance in both cases was very low, <70%, when considering the recommended fixed specificity of 80% to 95%. For patients with FC levels above 5- fold the cut-off level (>250μg/g), the post-test probability for active IBD was 85.7% for iChroma Calprotection and 64.1% for Buhlmann Quantum Blue assay. These results suggest that FC levels greater than 5 times the cutoff have a high level of accuracy and would be valuable in clinical decision making. We believe these results will help establish calprotectin testing in clinical laboratories. Clinicians may also apply different cutoff values depending on the purpose of the examination. However, these results are also dependent on the sample size and assay methods. Therefore, a larger cohort of the patient is recommended.

In the present study, we also evaluated the FC levels results of the two assays in relation to disease location in active UC patients. Of the 30 patients with active UC, 16 (53.3%) patients showed proctitis or proctosigmoiditis in colonoscopy and had significantly decreased FC levels of 12.3 (10.1–55.3) μg/g with iChroma Calprotectin assay. This is consistent with previous reports that proctitis and left-sided colitis tend to have lower FC levels compared to pancolitis [19–25]. These findings indicate that the two assays evaluated in the present study have relatively low sensitivities. This discrepancy should be investigated in the future using a larger cohort.

This study has several limitations. First, the sample size was relatively small and fecal characteristics were not taken into account. Second, we did not assess the FC levels in relation to disease activity or predicting the prognosis. Third, stool microscopy and culture to distinguish infectious enterocolitis were not available in most cases, which could be a potential confounder. In addition, we performed a head-to-head comparison of two POCT without ELISA, which is considered the gold standard test for fecal calprotectin. Last, we used both frozen or fresh samples, which could be a source of heterogeneity in the study. Further studies are necessary to confirm the results and to evaluate the usefulness of prognosis predictions in large cohorts.

This is the first study to evaluate the diagnostic performance of iChroma Calprotectin assay in comparison to Buhlmann Quantum blue using clinical samples. Based on our findings, ichroma calprotectin test, a new quantitative rapid lateral flow immunoassay, has had high potential for use in the clinical laboratory. The two rapid FC assays results showed a significant correlation, however, the two assays cannot be used interchangeably due to the unharmonized calibration. Overall, with optimized cut-off values, rapid FC tests could be a supportive test, in differentiating active IBD from inactive or organic bowel disease.

## Supporting information

S1 Table Fecal calprotectin levels of the study samples. (DOCX)
