# Supplementary material for: A comparison of diagnostic performance between two quantitative rapid fecal calprotectin assays in detecting active inflammatory bowel disease
Source: PLoS One. 2021 Aug 12;16(8):e0255974. doi: 10.1371/journal.pone.0255974 (PMC8360365; doi:10.1371/journal.pone.0255974)
Supplement: S1 Table — (DOCX) [file pone.0255974.s001.docx]

S1 Table. Fecal calprotectin levels of the study samples.

| NO. | Buhlmann Quantum blue | Ichroma calprotectin | Sex | Age | Classification | Diagnosis |
| --- | --- | --- | --- | --- | --- | --- |
| 1 | 99.9 | 9.9 | M | 41 | 1.UCA | Ulcerative colitis |
| 2 | 99.9 | 9.9 | M | 17 | 1.UCA | Ulcerative colitis |
| 3 | 99.9 | 9.9 | M | 67 | 1.UCA | Ulcerative colitis |
| 4 | 130 | 9.9 | F | 64 | 1.UCA | Ulcerative colitis |
| 5 | 141 | 9.9 | M | 32 | 1.UCA | Ulcerative colitis |
| 6 | 161 | 9.9 | M | 60 | 1.UCA | Ulcerative colitis |
| 7 | 142 | 10.04 | M | 43 | 1.UCA | Ulcerative colitis |
| 8 | 99.9 | 11 | M | 51 | 1.UCA | Ulcerative colitis |
| 9 | 130 | 12.21 | M | 34 | 1.UCA | Ulcerative colitis |
| 10 | 99.9 | 12.46 | M | 48 | 1.UCA | Ulcerative colitis |
| 11 | 99.9 | 13.93 | M | 40 | 1.UCA | Ulcerative colitis |
| 12 | 122 | 17.36 | F | 61 | 1.UCA | Ulcerative colitis |
| 13 | 120 | 17.89 | M | 38 | 1.UCA | Ulcerative colitis |
| 14 | 210 | 46.26 | M | 47 | 1.UCA | Ulcerative colitis |
| 15 | 1116 | 52.12 | M | 82 | 1.UCA | Ulcerative colitis |
| 16 | 304 | 61.04 | F | 50 | 1.UCA | Ulcerative colitis |
| 17 | 138 | 69.44 | F | 36 | 1.UCA | Ulcerative colitis |
| 18 | 99.9 | 81.54 | M | 24 | 1.UCA | Ulcerative colitis |
| 19 | 353 | 135.34 | M | 26 | 1.UCA | Ulcerative colitis |
| 20 | 112 | 149.64 | M | 28 | 1.UCA | Ulcerative colitis |
| 21 | 1293 | 228.64 | M | 63 | 1.UCA | Ulcerative colitis |
| 22 | 713 | 270.72 | M | 45 | 1.UCA | Ulcerative colitis |
| 23 | 300.1 | 352.96 | M | 45 | 1.UCA | Ulcerative colitis |
| 24 | 138 | 368.51 | F | 69 | 1.UCA | Ulcerative colitis |
| 25 | 1800.1 | 385.49 | M | 21 | 1.UCA | Ulcerative colitis |
| 26 | 1800.1 | 542.74 | M | 35 | 1.UCA | Ulcerative colitis |
| 27 | 1800.1 | 685.29 | M | 32 | 1.UCA | Ulcerative colitis |
| 28 | 1800.1 | 696 | M | 47 | 1.UCA | Ulcerative colitis |
| 29 | 458 | 842.27 | F | 53 | 1.UCA | Ulcerative colitis |
| 30 | 300.1 | 960 | M | 34 | 1.UCA | Ulcerative colitis |
| 31 | 99.9 | 9.9 | M | 44 | 2.UCI | Ulcerative colitis |
| 32 | 99.9 | 9.9 | M | 45 | 2.UCI | Ulcerative colitis |
| 33 | 99.9 | 9.9 | F | 70 | 2.UCI | Ulcerative colitis |
| 34 | 99.9 | 9.9 | M | 24 | 2.UCI | Ulcerative colitis |
| 35 | 99.9 | 9.9 | M | 21 | 2.UCI | Ulcerative colitis |
| 36 | 99.9 | 9.9 | F | 56 | 2.UCI | Ulcerative colitis |
| 37 | 99.9 | 9.9 | F | 39 | 2.UCI | Ulcerative colitis |
| 38 | 99.9 | 9.9 | M | 35 | 2.UCI | Ulcerative colitis |
| 39 | 99.9 | 9.9 | F | 33 | 2.UCI | Ulcerative colitis |
| 40 | 99.9 | 9.9 | F | 58 | 2.UCI | Ulcerative colitis |
| 41 | 99.9 | 9.9 | F | 43 | 2.UCI | Ulcerative colitis |
| 42 | 99.9 | 9.9 | M | 58 | 2.UCI | Ulcerative colitis |
| 43 | 99.9 | 9.9 | F | 43 | 2.UCI | Ulcerative colitis |
| 44 | 99.9 | 9.9 | F | 62 | 2.UCI | Ulcerative colitis |
| 45 | 99.9 | 9.9 | F | 36 | 2.UCI | Ulcerative colitis |
| 46 | 99.9 | 9.9 | M | 32 | 2.UCI | Ulcerative colitis |
| 47 | 114 | 9.9 | M | 58 | 2.UCI | Ulcerative colitis |
| 48 | 142 | 9.9 | F | 35 | 2.UCI | Ulcerative colitis |
| 49 | 182 | 9.9 | M | 61 | 2.UCI | Ulcerative colitis |
| 50 | 319 | 9.9 | M | 54 | 2.UCI | Ulcerative colitis |
| 51 | 181 | 12.37 | M | 37 | 2.UCI | Ulcerative colitis |
| 52 | 166 | 13.62 | F | 41 | 2.UCI | Ulcerative colitis |
| 53 | 99.9 | 15.92 | F | 33 | 2.UCI | Ulcerative colitis |
| 54 | 109 | 18.61 | M | 25 | 2.UCI | Ulcerative colitis |
| 55 | 184 | 18.73 | M | 32 | 2.UCI | Ulcerative colitis |
| 56 | 100 | 19.59 | M | 22 | 2.UCI | Ulcerative colitis |
| 57 | 502 | 19.87 | M | 49 | 2.UCI | Ulcerative colitis |
| 58 | 158 | 45.72 | M | 60 | 2.UCI | Ulcerative colitis |
| 59 | 171 | 51.8 | F | 42 | 2.UCI | Ulcerative colitis |
| 60 | 1480 | 53.22 | F | 41 | 2.UCI | Ulcerative colitis |
| 61 | 139 | 57.48 | M | 50 | 2.UCI | Ulcerative colitis |
| 62 | 99.9 | 68.48 | M | 71 | 2.UCI | Ulcerative colitis |
| 63 | 235 | 110.34 | M | 24 | 2.UCI | Ulcerative colitis |
| 64 | 140 | 113.8 | F | 21 | 2.UCI | Ulcerative colitis |
| 65 | 121 | 184.02 | M | 37 | 2.UCI | Ulcerative colitis |
| 66 | 1800.1 | 312 | M | 48 | 2.UCI | Ulcerative colitis |
| 67 | 1100 | 1000.1 | F | 53 | 2.UCI | Ulcerative colitis |
| 68 | 465 | 9.9 | M | 36 | 3.CDA | Crohn's disease |
| 69 | 183 | 11.92 | M | 44 | 3.CDA | Crohn's disease |
| 70 | 200 | 52.42 | M | 27 | 3.CDA | Crohn's disease |
| 71 | 813 | 90.4 | M | 20 | 3.CDA | Crohn's disease |
| 72 | 537 | 104.1 | F | 20 | 3.CDA | Crohn's disease |
| 73 | 1247 | 177.94 | F | 11 | 3.CDA | Crohn's disease |
| 74 | 482 | 259.3 | M | 19 | 3.CDA | Crohn's disease |
| 75 | 1800.1 | 354.86 | F | 18 | 3.CDA | Crohn's disease |
| 76 | 300.1 | 359.86 | F | 51 | 3.CDA | Crohn's disease |
| 77 | 631 | 540 | M | 53 | 3.CDA | Crohn's disease |
| 78 | 1800.1 | 579.15 | M | 23 | 3.CDA | Crohn's disease |
| 79 | 1560 | 599.74 | M | 14 | 3.CDA | Crohn's disease |
| 80 | 1800.1 | 1000.1 | M | 17 | 3.CDA | Crohn's disease |
| 81 | 1800.1 | 1000.1 | M | 17 | 3.CDA | Crohn's disease |
| 82 | 1800.1 | 1566.27 | F | 65 | 3.CDA | Crohn's disease |
| 83 | 110 | 13.4 | F | 21 | 4.CDI | Crohn's disease |
| 84 | 109 | 68.78 | M | 18 | 4.CDI | Crohn's disease |
| 85 | 99.9 | 9.9 | M | 19 | 5.IBS | IBS |
| 86 | 99.9 | 9.9 | M | 19 | 5.IBS | IBS |
| 87 | 99.9 | 9.9 | F | 29 | 5.IBS | IBS |
| 88 | 99.9 | 9.9 | M | 20 | 5.IBS | IBS |
| 89 | 99.9 | 9.9 | M | 47 | 5.IBS | IBS |
| 90 | 99.9 | 9.9 | M | 29 | 5.IBS | IBS |
| 91 | 99.9 | 9.9 | F | 21 | 5.IBS | IBS |
| 92 | 99.9 | 9.9 | M | 52 | 5.IBS | IBS |
| 93 | 99.9 | 9.9 | M | 20 | 5.IBS | IBS |
| 94 | 99.9 | 9.9 | F | 32 | 5.IBS | IBS |
| 95 | 99.9 | 9.9 | M | 32 | 5.IBS | IBS |
| 96 | 99.9 | 9.9 | M | 26 | 5.IBS | IBS |
| 97 | 99.9 | 9.9 | M | 27 | 5.IBS | IBS |
| 98 | 99.9 | 9.9 | M | 32 | 5.IBS | IBS |
| 99 | 99.9 | 9.9 | M | 30 | 5.IBS | IBS |
| 100 | 99.9 | 9.9 | F | 54 | 5.IBS | IBS |
| 101 | 99.9 | 9.9 | M | 15 | 5.IBS | IBS |
| 102 | 99.9 | 9.9 | M | 31 | 5.IBS | IBS |
| 103 | 99.9 | 9.9 | M | 30 | 5.IBS | IBS |
| 104 | 99.9 | 9.9 | F | 20 | 5.IBS | IBS |
| 105 | 99.9 | 9.9 | M | 28 | 5.IBS | IBS |
| 106 | 99.9 | 9.9 | F | 78 | 5.IBS | IBS |
| 107 | 99.9 | 9.9 | M | 19 | 5.IBS | IBS |
| 108 | 99.9 | 9.9 | F | 50 | 5.IBS | IBS |
| 109 | 99.9 | 9.9 | F | 26 | 5.IBS | IBS |
| 110 | 99.9 | 9.9 | M | 35 | 5.IBS | IBS |
| 111 | 99.9 | 9.9 | M | 17 | 5.IBS | IBS |
| 112 | 99.9 | 9.9 | M | 20 | 5.IBS | IBS |
| 113 | 99.9 | 9.9 | F | 53 | 5.IBS | IBS |
| 114 | 99.9 | 9.9 | M | 29 | 5.IBS | IBS |
| 115 | 99.9 | 9.9 | M | 80 | 5.IBS | IBS |
| 116 | 107 | 9.9 | F | 24 | 5.IBS | IBS |
| 117 | 156 | 9.9 | F | 74 | 5.IBS | IBS |
| 118 | 177 | 9.9 | M | 21 | 5.IBS | IBS |
| 119 | 99.9 | 10.43 | F | 53 | 5.IBS | IBS |
| 120 | 99.9 | 10.7 | F | 19 | 5.IBS | IBS |
| 121 | 132 | 14.78 | F | 21 | 5.IBS | IBS |
| 122 | 99.9 | 40.78 | M | 28 | 5.IBS | IBS |
| 123 | 193 | 41.2 | F | 63 | 5.IBS | IBS |
| 124 | 99.9 | 49.6 | M | 20 | 5.IBS | IBS |
| 125 | 305 | 50.28 | M | 23 | 5.IBS | IBS |
| 126 | 112 | 51.82 | M | 23 | 5.IBS | IBS |
| 127 | 148 | 86 | F | 67 | 5.IBS | IBS |
| 128 | 150 | 122.18 | F | 20 | 5.IBS | IBS |
| 129 | 99.9 | 9.9 | M | 45 | 6.other | Colon adenoma |
| 130 | 99.9 | 9.9 | F | 79 | 6.other | Colon adenoma |
| 131 | 99.9 | 9.9 | M | 71 | 6.other | Colon adenoma |
| 132 | 99.9 | 9.9 | M | 65 | 6.other | Colon adenoma |
| 133 | 122 | 9.9 | M | 82 | 6.other | Colon adenoma |
| 134 | 213 | 9.9 | M | 80 | 6.other | Colon adenoma |
| 135 | 99.9 | 10 | F | 72 | 6.other | Colon adenoma |
| 136 | 245 | 16.88 | M | 68 | 6.other | Colon adenoma |
| 137 | 420 | 17.67 | F | 68 | 6.other | Colon adenoma |
| 138 | 104 | 56.38 | F | 56 | 6.other | Colon adenoma |
| 139 | 167 | 106.1 | F | 59 | 6.other | Colon adenoma |
| 140 | 99.9 | 9.9 | M | 34 | 6.other | Enterocolitis |
| 141 | 99.9 | 9.9 | M | 61 | 6.other | Enterocolitis |
| 142 | 99.9 | 9.9 | F | 65 | 6.other | Enterocolitis |
| 143 | 99.9 | 9.9 | F | 67 | 6.other | Enterocolitis |
| 144 | 99.9 | 9.9 | F | 67 | 6.other | Enterocolitis |
| 145 | 101 | 9.9 | M | 68 | 6.other | Enterocolitis |
| 146 | 143 | 9.9 | M | 90 | 6.other | Enterocolitis |
| 147 | 154 | 9.9 | M | 34 | 6.other | Enterocolitis |
| 148 | 177 | 9.9 | M | 78 | 6.other | Enterocolitis |
| 149 | 189 | 9.9 | M | 58 | 6.other | Enterocolitis |
| 150 | 228 | 9.9 | F | 90 | 6.other | Enterocolitis |
| 151 | 386 | 9.9 | M | 25 | 6.other | Enterocolitis |
| 152 | 225 | 15.93 | F | 79 | 6.other | Enterocolitis |
| 153 | 118 | 16.4 | M | 75 | 6.other | Enterocolitis |
| 154 | 222 | 18.13 | F | 89 | 6.other | Enterocolitis |
| 155 | 138 | 19.24 | M | 26 | 6.other | Enterocolitis |
| 156 | 136 | 43.7 | M | 35 | 6.other | Enterocolitis |
| 157 | 303 | 113.46 | M | 22 | 6.other | Enterocolitis |
| 158 | 292 | 263 | F | 48 | 6.other | Enterocolitis |
| 159 | 99.9 | 9.9 | F | 86 | 6.other | GI bleeding |
| 160 | 99.9 | 9.9 | F | 78 | 6.other | GI bleeding |
| 161 | 99.9 | 9.9 | F | 44 | 6.other | GI bleeding |
| 162 | 129 | 9.9 | F | 72 | 6.other | GI bleeding |
| 163 | 99.9 | 9.9 | M | 80 | 6.other | GI ulcer |
| 164 | 99.9 | 9.9 | F | 66 | 6.other | GI ulcer |
| 165 | 99.9 | 11.85 | M | 43 | 6.other | GI ulcer |
| 166 | 383 | 12.51 | M | 56 | 6.other | GI ulcer |
| 167 | 224 | 58.02 | M | 82 | 6.other | GI ulcer |
| 168 | 99.9 | 9.9 | M | 55 | 6.other | None specific GI symptom |
| 169 | 99.9 | 9.9 | M | 78 | 6.other | None specific GI symptom |
| 170 | 99.9 | 9.9 | F | 61 | 6.other | None specific GI symptom |
| 171 | 99.9 | 9.9 | M | 58 | 6.other | None specific GI symptom |
| 172 | 99.9 | 9.9 | M | 68 | 6.other | None specific GI symptom |
| 173 | 99.9 | 9.9 | M | 71 | 6.other | None specific GI symptom |
| 174 | 99.9 | 9.9 | F | 47 | 6.other | None specific GI symptom |
| 175 | 99.9 | 9.9 | F | 87 | 6.other | None specific GI symptom |
| 176 | 99.9 | 9.9 | M | 88 | 6.other | None specific GI symptom |
| 177 | 99.9 | 9.9 | F | 39 | 6.other | None specific GI symptom |
| 178 | 160 | 9.9 | F | 29 | 6.other | None specific GI symptom |
| 179 | 272 | 9.9 | F | 75 | 6.other | None specific GI symptom |
| 180 | 454 | 9.9 | F | 49 | 6.other | None specific GI symptom |
| 181 | 99.9 | 10.24 | M | 79 | 6.other | None specific GI symptom |
| 182 | 99.9 | 10.91 | F | 17 | 6.other | None specific GI symptom |
| 183 | 99.9 | 11.13 | M | 35 | 6.other | None specific GI symptom |
| 184 | 250 | 12.39 | F | 81 | 6.other | None specific GI symptom |
| 185 | 99.9 | 13.03 | F | 67 | 6.other | None specific GI symptom |
| 186 | 99.9 | 40.9 | M | 80 | 6.other | None specific GI symptom |
| 187 | 132 | 52.46 | F | 37 | 6.other | None specific GI symptom |
| 188 | 143 | 53.92 | M | 70 | 6.other | None specific GI symptom |
| 189 | 245 | 57.68 | F | 86 | 6.other | None specific GI symptom |
| 190 | 127 | 58.56 | F | 67 | 6.other | None specific GI symptom |
| 191 | 99.9 | 66.04 | F | 73 | 6.other | None specific GI symptom |
| 192 | 152 | 156.6 | M | 86 | 6.other | None specific GI symptom |
